# Plant-Produced Monoclonal Antibody as Immunotherapy for Cancer

**DOI:** 10.1155/2020/3038564

**Published:** 2020-08-24

**Authors:** Meher Un Nessa, Md. Atiar Rahman, Yearul Kabir

**Affiliations:** ^1^Environmental Science Discipline, Khulna University, Khulna 9208, Bangladesh; ^2^Department of Biochemistry and Molecular Biology, University of Chittagong, Chittagong 4331, Bangladesh; ^3^Department of Biochemistry and Molecular Biology, University of Dhaka, Dhaka 1000, Bangladesh

## Abstract

Plant-based products have expanded to include cancer immunotherapy, which has made great strides over recent years. Plants are considered inexpensive and facile production platforms for recombinant monoclonal antibody (mAb) due to the latest advancements and diversification of transgenic techniques. Current human biologics, including those based on mAbs produced by fermentation technologies using primarily mammalian cell cultures, have been replaced by plant-produced mAbs, which are cost effective, more scalable, speedy, versatile, and safer. Moreover, the use of animals for antibody production is always a question of ethical unambiguity, and the suitability of animal models for predicting the immunogenicity of therapeutic mAbs in humans and transposition of the immunogenic potential of therapeutic antibodies in animals to the human situation has no scientific rationale. Quite a few plant-based mAbs are approved for the treatment of cancer, ranging from tumors to hematological malignancies. This review focuses on the cutting-edge approaches for using plant-derived mAbs to suppress or prevent cancers. It also discusses the avenues taken to prevent infection by oncogenic viruses, solid tumors, lymphomas, and other cancerous conditions using mAbs. The review emphasizes the use of a plant-derived monoclonal antibody as a premier platform to combat cancer.

## 1. Introduction

Cancer remains one of the most feared diseases of our time because of its ability to metastasize, our failure to manage and treat the disease properly, and lack of a complete understanding of cancer development mechanisms. Despite the significant improvement in cancer therapies over the last three decades and applying molecular biology techniques, the global burden of cancer continues to increase. It is still one of the most devastating diseases worldwide. Until recently, the basic strategies to treat cancer included chemotherapy, surgery, and radiation therapy, and very recently, targeted therapy either in combination or in sequence has been introduced to reduce, remove, eliminate, or alleviate tumors. Although these strategies provide efficacy and effective actions in making enduring relief in patients with nonmetastatic and early cancers, they are usually unsuccessful in producing long-lasting benefits in patients with late-stage disease, except in certain leukemias, lymphomas, germ cell tumors, and testicular carcinomas. Unfortunately, these multidimensional approaches often face two main problems: development of resistance related to lengthy use and the presence of severe adverse effects made by the overall doses of radiation and the significant impact of cytotoxic agents on healthy cells and physiologic functions [1]. Apart from these, the economic burden from huge expenses for cancer treatments imposes an extreme social burden on a cancer patient and their family members. In this context of increasing cancer prevalence and socioeconomic burden, an alternative cancer-preventing therapeutic option is indispensably urgent. Immunotherapy has blossomed as a treatment option with enormous potential to avert or suppress cancer progression due to its direct influence on malignant cells with superior efficiency to target and attack the cancer cells [2]. However, recent studies on antibody-mediated killing responses against tumor cells and the generation of a huge number of antibodies against antitumor response helped produce monoclonal antibody (mAb) for recognizing specific antigens available on the surface of cancer cells. Despite their advantages and therapeutic potential, some functional limitations, such as the absence of cost-effective manufacturing of mAbs with high quality and purity level and limited scalability of the mammalian production system, impaired their widespread application as therapeutic agents for cancer. Additionally, mAb produced by *in vivo* methods can contain various mouse proteins and other contaminants that could lead to humans harboring pathogens. Therefore, for therapeutic applications, heterogeneous production platforms with affordable costs, scalability, and safety have been ensured using other bioorganisms such as plants, insects, bacteria, and yeast [3]. Among them, plants offer a novel system for the development and production of a monoclonal antibody with the criteria described above of expression paradigms.

## 2. Materials and Methods

A comprehensive literature work has been pursued to gather relevant information from major science repositories, including Google Scholar, Medline, PubMed, and Science Direct, specifically to classify plant-based monoclonal antibodies and their potential for different forms of cancer. Some articles were cited from other publications or accessed directly through the journal's website. They were considered based on the geographical region of their origin. The literature published in recent years is highly preferred for relevant actions. The keyword combinations for the search were plant-produced monoclonal antibody, monoclonal antibodies for cancer, plant-based monoclonal antibodies, monoclonal antibodies from a plant in cancer, plant products as monoclonal antibodies, etc. Additional information was incorporated by using some other keyword combinations such as plant extract for cancer, medicinal uses of plant-based monoclonal antibodies, and phytomedicinal monoclonal antibody. A total of 53 research articles reporting the action of plant-produced monoclonal antibodies in cancer have been recovered and presented in this review.

### 2.1. Scopes for the Readers/Audience

This review extends the novice researchers' scope to identify research gaps between the existing line of therapeutics and prospective alternatives but using a highly safer option for cancer treatment with monoclonal antibodies produced from plant bioresources. The decision-makers will be able to have the ways for producing, formulating, and marketing the plant-based mAbs to create easy access for those desiring the category of drugs described above. As a whole, the researchers will be able to focus on the current plant expression systems offering the features beyond the traditional benefits of eukaryotic protein modification and high adaptability, such as low cost and increased safety. An enormous opportunity is expected to develop with novel transient expression vectors that allow mAbs to be produced at unprecedented speed to mitigate potential pandemics. The potentiality for glycoengineering with plants to make mAbs with unique mammalian glycoforms for newer utility in making safe biobetters will also be exposed.

### 2.2. Why Immune Therapy Is Different: Scientific and Clinical Basis

Cancer is mostly a process of abnormal cell proliferation in an uncontrolled manner, and the cells usually fail to die normally. Cancer cells can invade healthy tissues and organs and eventually extend over the body's parts through the blood and lymphatic systems. In order for altered cells to be established in a host, they should develop ways to avoid eradication by the immune system and respond to treatment. Manipulation of the therapeutic approach can substantially alter the innate immune system, which leads to cell death and, under a suitable physiological environment, adaptive and innate immunity leading to oncolysis while helping long-term memory responses. Thus, immunotherapy for cancer is a treatment that allows a patient's immune system to fight cancer [1].

Conventional chemotherapy desires to rapidly attack the dividing cells within the body through unrestrained, static, indiscriminate, and toxic direct attack on both the malignant and normal cells to destroy the cancer cells more than it destroys the host cells. Recently, it has been reported that this may also target both stromal cells and immune cells [4, 5]. On the contrary, the salient features of immune-based therapy include the breadth of response, specificity, and memory. The tumor immunotherapies usually engage the immune system to identify and eliminate tumor cells, rather than targeting cancerous cells directly, and thus offers a more effective alternative treatment for some cancer patients. Unlike other conventional therapies, immune therapy may take a longer time to get the feedback of treatment because the immune system is mobilized to attack tumors. Sometimes, tumors may even show some pseudoprogression where the tumors grow at first, but eventually, they swell in size due to the infiltration of immune cells into tumors [6]. Most importantly, if immunotherapy can successfully activate the immune memory, it allows the body's defense against a threat to continue even after therapy has been withdrawn and possibly for as long as the patient's entire lifespan [1].

Since immunotherapy is based on the immune system's natural power to identify and remember cancer cells, it can be active against all types of cancer. It has been accepted as the first line of treatment for several cancers. Its efficiency was also confirmed against several types of cancers that were factually resistant to chemotherapy and radiation treatments [6]. Cancer cells share many resemblances with the healthy host cells, and this offers a challenge for achieving high levels of selective cytotoxicity. Chemotherapeutic monoclonal antibodies have appeared as standard therapeutic agents for many cancers in humans in the past decade; they were engineered with the predicted advantage of specificity, thus acting as “targeting missiles” toward cancer cells [7].

### 2.3. Monoclonal Antibodies: A New Subway to Prevent Cancer

The immune system is a sophisticated host defense system consisting of many biological structures and processes that identify and destroy harmful disease-causing substances, such as bacteria and viruses. The immune system uses the immunoglobulins (Ig) or antibodies as powerful molecular tools to recognize and neutralize minute quantities of a given target analyte [8]. Recently, the mechanisms of antibody-mediated killing responses against tumor cells that induce consistent, effective, and durable cancer-suppressing activities have been revealed by experimental and clinical studies [3].

From 1940, the scientific understanding of antibodies such as its generation, diversity, and structure and Brunet's clonal selection theory demonstrated that one cell produces one specific antibody [9]. Further, monoclonal antibodies (mAbs) generated using hybridoma technology or fusion of spleen cells from an immunized mouse with immortalized myeloma cells [10, 11] can be used against one specific epitope. Researchers are now designing mAbs that can be targeted at a specific antigen found on cancer cells. These monoclonal antibodies can serve as substituted antibodies that can restore, boost, or imitate the immune system's attack on cancer cells. They are intended to bind to antigens that are usually more abundant on cancer cells' surfaces than on healthy cells' surfaces [12, 13]. For cancer, selecting the right antigen is not always easy, and until now, mAbs have demonstrated to be more suitable against some cancers than against others. As more antigens associated with cancer are found, researchers have created mAbs against more and more cancers, and these mAbs are now under clinical trials on several kinds of cancers. In the last ten years, record numbers of antibody therapeutics have entered into clinical studies and have been approved, and over 570 antibody therapeutics are ongoing in various clinical phases; 62 of them are currently in late-stage clinical studies. Depending on the therapeutic area, the progress of some clinical studies from phase 1 to approval has seen favorable success rates, ranging from 17 to 25% [14].

**Figure 1:**
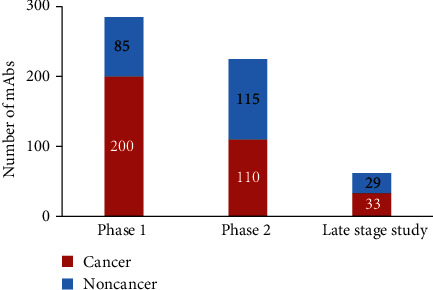
Clinical phases for antibody therapeutics in development. Totals include only antibody therapeutics (biosimilars and Fc fusion proteins were excluded) sponsored by commercial firms [14].

Even though advances in basic research guided the development of hybridoma technology in 1975, the first mAb used as a therapeutic was approved after eleven years. Since then, the development of mAbs has played a significant role in the pharmaceutical industry [15]. The US Food and Drug Administration (FDA), in the last couple of decades, has approved more than a dozen of mAbs as therapeutics for hematological malignancies and solid tumors [16]. The achievement of antibody therapeutics has inspired pharmaceutical companies to participate in the development of these molecules. Jointly, pharmaceutical industries are presently supporting the clinical trials of more than five hundred seventy mAbs. Ninety percent of those are going through early-stage interventions intended to evaluate the safety (Phase 1) or safety and preliminary efficacy of the molecules in different patient populations, as shown in Figure 1. Among Phase 1 mAbs, most (~70%) are for cancer, reflecting the recent significant increase in the entrance of anticancer antibodies in clinical studies. In contrast, at Phase 2 and end-stage clinical studies, the number of mAbs being planned for the treatment cancer is similar to the number of mAbs being planned for the treatment of noncancer diseases [14].

### 2.4. mAb Structure and Anticancer Mechanism

Antibodies are mostly figured as the typical mammalian serum-type immunoglobulin G (IgG), which contains two identical light chains and two identical heavy chains and linked by disulfide bonds. An antibody is made up of a constant region, the region that has a constant structure for different antigens, and a variable region, the region that changes to different structures depending on differences in antigens. The amino acid terminal sequence domains of the heavy and light variable chains are called V_H_ and V_L_, respectively. In contrast, the corresponding constant sequence domain of each chain is called C_H_ and C_L_. These variable and constant regions are dedicated to preventing pathogens from entering or damaging cells and recruiting various immune-related molecules and cells to disrupt antigen's functions and destroy tumor cells or pathogens through binding with the specific antigen. The antigen-binding fragment (Fab) is the region that binds to Fc, the fragment crystallizable region or the tail region of an antibody, and interacts with cell surface receptors (Fc receptors) and some proteins of the complement system. The Fc regions of IgG bear a highly conserved N-glycosylation site, which is inevitable for Fc receptor-mediated activity [3, 17].

**Figure 2:**
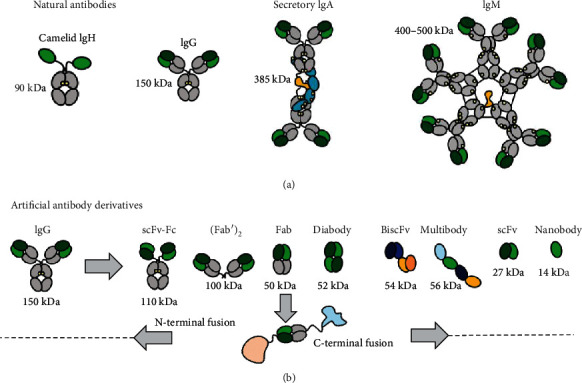
Domain architecture of natural antibodies and some engineered recombinant variants. Domains representing the antigen-binding site are indicated in green and the constant domains in gray [18].

Antibodies are the immunoglobulins classified into five groups: IgM, IgG, IgA, IgD, and IgE, according to their structural, physiochemical, and immunological properties. These five classes have different Fc regions and thus different effects or functions, and some of these have more complex structures than the simplest IgG. For example, IgA (sIgA), the secretory-type, has four heavy chains and four light chains, which assemble as two IgG-like tetramers and a secretory component and a joining chain. The expression of a full-sized IgG in plants requires two genes, whereas the expression of a sIgA requires four genes. In addition to natural antibodies, there are also many smaller antibody derivatives that are functional in terms of antigen binding such as Fab, F(ab′)_2_, minibodies, large single chains, single-chain variable fragments (scFvs), bispecific scFvs, diabodies, camelid antibodies, nanobodies, and antibody fusion proteins as shown in Figure 2. The functional and structural differences between variable domains, constant domains, and the free N- and C-terminal ends of the individual domains have given rise to enormous modifications, derivatives, and combinations [18]. Although antibodies are not a natural production of plants, plants can be engineered to do so by introducing the corresponding immunoglobulin genes. In this way, plants can be instructed to produce antibodies in all kinds of different formats that can target the antigen of choice.

Chemotherapeutic monoclonal antibodies are designed to function in diverse ways. A particular drug may work by more than one means. They can act by directly attacking cancer cells, flagging cancer cells, and thus binding cancer and immune cells, triggering cell-membrane destruction, blocking cell growth, preventing blood vessel growth, blocking immune system inhibitors, delivering radiation treatment, and delivering chemotherapy. They can target cancer cells by binding to cell surface antigens. Cell surface antigens include antigens associated with growth and differentiation, such as cluster of differentiation (CD: e.g., CD20, CD30, CD33, and CD52), epidermal growth factor receptor (EGFR), carcinoembryonic antigen (CEA), human epidermal growth factor receptor 2 (HER2), receptor activator of nuclear factor kappa-B ligand (RANKL), vascular endothelial growth factor (VEGF), VEGF receptor (VEGFR), integrins (e.g., *α*V*β*3 and *α*5*β*1), fibroblast activation protein (FAP), and extracellular matrix metalloproteinase inducers (EMMPRIN) [7].

The cancer cell-neutralizing capability of antibodies is considered to be a critical mechanism for immunotherapy. However, certain additional activities, such as antibody-dependent cellular cytotoxicity, antibody-dependent cellular phagocytosis, and complement-dependent cytotoxicity, are developing as important contributors to the protection of immunity. Antibody effector functions are due to the effective interactions between their Fc region and Fc receptors of immune cells, including the binding property of mAb to the targeted antigens [3]. In cancer treatment, antibodies have been exploited to eliminate tumor cells by blocking signaling pathways. Recent achievements in monoclonal therapeutic strategy have been attained through technological breakthroughs that enable the creation of modified antibodies that display greater abilities to recruit innate immune killing through antibody Fc glycosylation modification. This posttranslational alteration of the Fc domain of antibodies *in vivo* could accelerate the antibodies' therapeutic efficacy to design monoclonal therapeutics and next-generation vaccines [19].

### 2.5. Classification of Chemotherapeutic Monoclonal Antibody (CmAb)

Monoclonal antibodies (mAbs) are now well known as targeted therapeutics for malignancies, transplant rejection, and infectious and autoimmune diseases, besides a range of new indications. However, the administration of mAbs can cause four kinds of risks: autoimmune diseases, cytokine release syndrome, opportunistic infections, and organ toxicity. Depending on the degree of humanization, which is highly variable, immunogenicity could also lead to adverse effects due to immune-complex formation [20, 21]. Thus, one significant attention of antibody engineering in the past thirty years has been to decrease immunogenicity and enhance the production of antibodies appropriate for human therapeutics. With the understanding of disease biology, recombinant engineering technology, and antibody mechanisms, a range of novel types of antibody molecules are evolving as promising new generation therapeutics [11].

**Figure 3:**
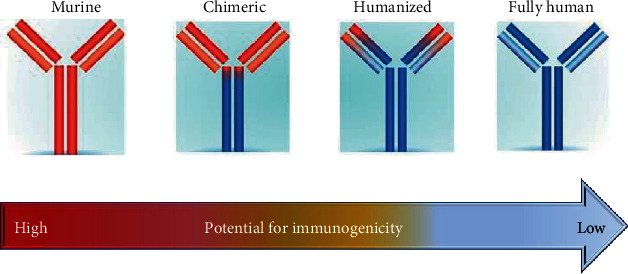
Classification of chemotherapeutic monoclonal antibodies and their corresponding immunogenicity.

Advances in genetic engineering technologies have resulted in the development of four main types of CmAbs: murine, chimeric, humanized, and human CmAbs; these were derived exclusively from mouse and were the first to be applied in cancer chemotherapeutics. Chimeric CmAbs typically comprise variable regions gleaned from a murine source, and constant regions (65%) from a human source can also be nonhumanized (chimeric trifunctional CmAbs), such as the rat-mouse hybrid. Humanized CmAbs are predominantly (90%) engineered from a human source except that the Fab portion's complementarity-determining regions are of murine origin [7]. Chimeric trifunctional CmAbs are characterized by a unique capacity to bind with three different cell types: tumor cells, T lymphocyte cells, and accessory cells; thus, they transiently link immune effector cells to tumor cells, which produce cellular cytotoxicity toward the tumor cells [22, 23]. The development of chimeric CmAbs that possess a fully human Fc portion provided considerably less immunogenic and more efficient interaction with human effector cells and the complement system than murine CmAbs. Humanized CmAbs are even less immunogenic than chimeric CmAbs. Human CmAbs, which are 100% human, are engineered from transgenic mice. Compared to chimeric and humanized CmAbs, they have higher affinity values toward human antigens and minimal or no hypersensitivity responses, as explained in Figure 3 [7, 24].

Chemotherapeutic monoclonal antibodies may be conjugated to other forms of cancer therapy, and this conjugation provides a targeted attack on cancer cells and therefore reduced widespread systemic toxicities to healthy cells. There are three types of conjugated CmAbs: radiolabelled CmAbs (linked to radionuclide particles), chemolabelled CmAbs (linked to antineoplastic drugs), and immunotoxin CmAbs (linked to plant and bacterial toxins) [7]. Antibody-drug conjugates (ADCs) are among the fastest-growing groups of cancer therapeutics. More than 60 ADCs are in clinical development for cancer therapy [25].

ADCs characteristically target overexpressed and internalizing antigens on the cancer cell surface and in solid tumors, so that a drug is released very selectively in the tumor microenvironment [26]. A perfect cytotoxic agent should be extremely effective, remain stable while connected to ADCs, destroy the targeted tumor cell upon internalization and release from the ADCs, and uphold its activity in multidrug-resistant tumor cells [27].

### 2.6. Advantages of Using the Plant for mAb Production

Despite significantly effective therapeutic actions, mAb treatment for cancer has not been established due to the high production expenditures, potential human pathogen contamination, and limited expandability of the mammalian cell-mediated system. Consequently, heterologous production platforms with safety, cost effectiveness, and scalability were established using different organisms such as bacteria, insects, yeast, and plants [3]. Although the existing fermenter-based production of antibodies has biomedical importance, it is costly and tedious and has low yield obtained via the purification process. Plants can be used as promising biofactory systems for the large-scale antibody production to defeat cancer and can bring hope for resource-poor nations to make a move from concept to reality. Some are already approved by the FDA for a clinical trial (Table 1). This is due to the high production capacity, inexpensive large-scale cultivation process, low downstream processing requirements that can be grown under containment conditions, and avoidance of ethical issues associated with transgenic animals [11, 28]. Currently, there are over a dozen FDA-approved mAbs, and as many as 700 therapeutic Abs may be under development. As of 2001, four antibodies expressed in plants had shown the potential to be useful as therapeutics. A chimeric secretory IgG/IgA antibody effective against a surface antigen of *Streptococcus mutans* has been expressed in tobacco and has been demonstrated to be effective against dental caries. Therefore, plants have potential as a virtually unlimited source of mAbs, referred to by some as “plantibodies.” Tobacco plants have been used extensively for antibody expression systems [17]. However, several other plants have been used, including potatoes, soybeans, alfalfa, rice, and corn. Antibody formats can be full sized, Fab fragments, single-chain antibody fragments, bispecific scFv fragments, membrane-anchored scFv, or chimeric antibodies. Plant cells, unlike mammalian cell expression systems, can express recombinant secretory IgA (sIgA).

Compared to using mammalian cells in conventional methods, the use of plants for Ab production proposes numerous irreplaceable benefits. Plants are widespread, plentiful, and develop rapidly; they typically mature after one season of growth. It is possible to bring the product to the market within a short time, which eventually lowers the production cost. Plants also reduce screening costs for bacterial toxins, viruses, and prions because they are less likely to introduce human or animal pathogens than mammalian cells or transgenic animals [29]. It has been reported that plant-based antibodies expressing up to 1% of total soluble protein will cost 0.1% of that of the mammalian cell culture system and up to 2-10% of microbial systems [30]. Plants share a comparable endomembrane system and secretory pathway with human cells, distinct from bacterial and other prokaryotic systems. Although plants produce a reasonably high yield of Abs in a comparatively shorter time, they do not activate immune responses that animal Abs are prone to do after facing foreign/non-self-agents [31].

Like animal cells, plant cells also have posttranslational modification mechanisms that allow them to be considered factories for therapeutic proteins, including antibodies [32]. It has been reported that glycoengineered plants have a considerably higher degree of glycan homogeneity. For example, the plant-derived version of h-13F6, an anti-Ebola virus monoclonal antibody carrying the complex N-glycosylation and lacking a fucose core, showed higher efficacy than the original version originating from mammalian cells [11]. Furthermore, plants are proficient in synthesizing and assembling all types of Ab molecules effectively, for example, the tiniest antigen-binding domains, fragments, and full-length and even multimeric Abs [29]. Therefore, among all kinds of production platforms, the use of plants to produce anticancer mAbs is now attracting attention.

### 2.7. Posttranslational Glycosylation in Plants

Posttranslational alterations of proteins happening in plant cells resemble those in animal cells. The accurate assembly of complex molecules, such as Abs, is supported by chaperones that facilitate the folding and formation of disulfide bonds. At the same time, the addition of *N*-glycans is accomplished by particular cellular glycosyltransferases [33]. There is a significant difference between mammalian and plant-derived glycoproteins because N-glycan synthesis in the endoplasmic reticulum is almost well preserved in all eukaryotic cells. In contrast, N-glycan processing and O-glycan biosynthesis in the Golgi apparatus are kingdom specific [34]. A major concern is the presence of beta 1,2-xylose and core alpha 1,3-fucose residues on complex N-glycans, as these nonmammalian N-glycan residues may inflame undesirable side effects in humans [34–36]. This highlights the need for the use of glycoengineered plants to remove any potentially antigenic N-glycan structures, if there is, for the production of plant-derived recombinant proteins intended for parenteral human application due to possible immunogenic reactions. Plants' fast, flexible, and easily scalable endogenous N-glycosylation machinery permits the synthesis of complex N-glycans lacking *β*-1,2-xylose and core *α*-1,3-fucose. Therefore, the removal or distraction of the genes responsible for integrating these glycan epitopes, i.e., 1,2-xylosyltransferase and core 1,3-fucosyltransferase, offers a sophisticated technique to solve this issue [36]. It is also evident that the glycoengineered plant-derived antibodies, e.g., 2G12 and its Chinese hamster ovary (CHO) cells, have performed and produced counterparts [37]. The feasibility of this strategy was confirmed by the generation of *Arabidopsis thaliana* knockout plants lacking XylT and 1,3-FucT. Without showing any noticeable phenotype under standard growth conditions, these plants were viable and produced proteins carrying complex N-glycans lacking xylose and fucose [36].

The final and most complicated step of human N-glycosylation is terminal sialylation. Several drugs need sialylated oligosaccharides for optimal therapeutic potency, whereas convincing evidence suggests that plants do not have sialylated glycoproteins. Until recently, only mammalian cell-based systems were used for manufacturing, which can accomplish this vital posttranslational modification. The introduction of six proteins from the mammalian sialylation pathway into plants permits the biosynthesis of sialic acid, its activation, its transport into the Golgi apparatus, and finally its transfer onto terminal galactose, which is a milestone in plant glycoengineering [38]. This shows the massive flexibility of plants, allowing them to tolerate mammalian glycosylation and the large extent of conservation between mammals and plants [36]. Overall, current advances in plant glycoengineering permitted the synthesis of “human-like” recombinant glycoproteins with a highly homogeneous glycosylation pattern. Thus, plant expression systems are considered resourceful platforms for the production of mAbs with enhanced desired features [33].

### 2.8. Production of mAbs in Transgenic Plants

With current progress in genetic engineering, researchers now produce transgenic plants with multiple ideal traits. They are now capable of inserting advantageous/desired genes from a completely different species or even from a different kingdom into the target plant [39]. These transgenic plants are considered one of the most promising human therapeutic Ab syntheses [11]. The first correctly assembled and functional human antibody, IgG, was successfully produced in transgenic tobacco plants and dates back to almost 30 years ago [40]. This initial success was quickly followed by the successful expression of different antibody formats such as secretory immunoglobulin A (sIgA), Fab fragments, single-chain antibody fragments (scFvs), minibodies, single variable domains, antibody fusion proteins (immune cytokines), scFv-Fc antibodies, and camelid heavy-chain antibodies also in transgenic plants [33, 41]. Twenty years after the successful expression of sIgA antibodies in transgenic plants, the multimeric antibody IgM was recently produced in plants [42].

There are two types of expression strategies based either on the stable transformation of the nuclear genome or on transient expression systems exploiting viral or *Agrobacterium tumefaciens* transfer DNA (T-DNA) expression vectors. Transgenic plants producing correctly assembled whole mAbs were conventionally obtained by cross-pollinating two transgenic lines separately transformed with the antibody heavy-chain (HC) or light-chain (LC) genes. This time-consuming strategy is now replaced by more proficient and fast approaches based on binary vectors containing HC and LC coding sequences in the same T-DNA. Thus, complete IgGs can be produced from transgenic plants in a single transformation event [43]. In the case of transient or epichromosomal transformation, the introduced sequence is not heritable; this is essentially a batch process. This reduces the risk of environmental biosafety issues linked to the propagation of the transgene through seeds or pollen. Generally, large amounts of protein were produced through this approach in a very short period, usually a few days to weeks, which is not achievable via stable transformation [33]. As every plant in each batch should be infiltrated with bacteria, the potential drawback of this rapid method for massive scale production is that this infiltration merely transfers the fermentation costs of bacteria production [44]. The use of transgenic plants can overcome the problem through a well-defined master and working seed banks that can be established. Consecutive batches can be used without further manipulation [45]. These make transgenic plants the most suitable plant-based arrangement for very-large-scale production. Greenhouses or vertical farming units appear to be the most prospective scenarios for the mass production of mAbs in transgenic plants, whereas, due to the absence of containment, an open field approach for the cultivation of transgenic plants may not be able to ensure the complete biosafety for such pharmaceutical products [46].

Other types of production arrangements are plant-based but require cultivation systems similar to those used with mammalian cell cultures, such as aquatic plants, plant cell suspension cultures, and plant tissue cultures such as hairy root culture. These denote an effective method for heterologous protein synthesis in a sterile condition with low contamination risks by human components and pathogens. Additionally, both transformed plant cells and organs can be propagated indefinitely with a simple nutrient requirement. The protein of interest can be secreted into the culture medium, thus providing easy product collection and purification. On the other hand, the low protein yields (in the range of mg per liter of culture) and the complications in setting up large-scale production in bioreactors represent the foremost challenges for the future exploitation of these plant expression platforms [33, 46].

### 2.9. Plant Selection for Antibody Production

There are numerous plant species, which can be proficiently engineered for mAb production. These include *Nicotiana benthamiana* (a related wild species of tobacco), *Arabidopsis thaliana*, lettuce, potato, and maize; however, a significant amount of antibodies stated in the literature have been expressed in transgenic tobacco (*N. tabacum*) [33]. The high biomass yield and the rapid scale-up by high-volume seed production are the main advantages of tobacco compared to other plant species. The whole tobacco plant biomass (both leaf and stem) can produce the recombinant therapeutic proteins, eventually increasing the upstream production cost effectively [47]. Additionally, tobacco is a nonfood, nonfeed, and well-specified expression system, excluding human pathogen contamination, which can decrease biosafety concerns. The other leafy plant alfalfa (*Medicago sativa*) has a high yield of biomass and a homogeneous glycan structure, making it attractive for antibody production and giving it a comparative advantage over the tobacco plant [48]. Tobacco contains nicotine or other toxic alkaloids that require an additional extraction procedure, and tobacco produces heterogeneously N-glycosylated antibodies. Oxalic acid compounds remain in alfalfa that affect the downstream processing and produce lower amounts of leaf biomass than tobacco, which is the major disadvantage of alfalfa. At the same time, this problem is overcome by the high level of protein in alfalfa leaf tissues, which maximizes the accumulation of recombinant antibodies in plant biomass. However, alfalfa is used as an animal feed though the biosafety concern is not resolved [11]. Some vegetable plants which have comparatively high total soluble protein levels might be advantageous to use for recombinant protein expression. Compared to other plants, the leaf biomass of Chinese cabbage has the highest total soluble protein level, making it a potential candidate bioreactor for the production of recombinant therapeutic proteins [3]. The seeds of legumes and cereal crops such as soybean, rice, and maize have been used for antibody production. Although maize is favored because of its inexpensive, high-quality, large-scale output, it is a wind-pollinated species; thus, it contains the risk of outcrossing to food crops. As seeds have a low level of protease activity due to the high level of protease inhibitors in most of the cases, antibodies expressed in corn seeds are stable for more than three years without losing activity at room temperature [49, 50], whereas the significant drawback of leafy crops is that specific proteins are unstable unless the leaf tissue is frozen or processed because leaves with an active metabolism have high protease activities for degrading particular proteins [51]. Even though rice is a food crop, it has strange advantages over other plants. Transgenic rice seeds have been developed for the production of a delivery vehicle for oral tolerogens [52]. Exciting results have also been obtained in the small aquatic plant *Lemna minor* (duckweed) [53].

Although the plants show potential advantages in antibody production, they may have allergic reactions to plant proteins which is what human N-glycosylation is incapable of; i.e., their culture parameter becomes uncontrollable, and contamination risks for soil, bacterium, and pollen are less avoidable [11]. Besides, as plants and prokaryotes have differences in codon usage patterns, this can lead to the inefficient expression of prokaryotic proteins in plants [17]. The concern over possible toxin transmission to food crops can be significantly reduced by growing plants in contained spaces such as greenhouses. Although antibodies have been described to have numerous benefits, there are concerns that the purity of food crop strains could be at risk since plants carrying antibodies could contaminate food crops or toxins from pesticides or fertilizers could be transmitted to other plants. Therefore, it has been suggested that the plants that are not used as food for people or feed for livestock should be utilized to produce antibodies [29]. Since each plant species has its own physical and physiological characteristics affecting the expression and glycosylation of recombinant glycoproteins, careful consideration in the selection of plant species is a must for the successful production of antibodies.

## 3. Conclusion and Future Prospects

The plant-produced monoclonal antibody-based immunotherapy helps the immune system recognize and target cancer cells, and it is hoped that it can be made into a versatile answer to cancer. Recent success and advancement in the designing of monoclonal therapeutics have been achieved through a technological quantum leap that facilitates the generation of modified antibodies capable of exhibiting enhanced potentials, reduced immunogenicity, and reduced widespread systemic toxicities to healthy cells. Enormous progress has been shaped in recent years in the field of plant-made mAbs. Plants could be engineered for introducing expected immunoglobulin genes because they do not naturally produce antibodies. With the aid of new genetic engineering tools, researchers insert the desired gene(s) into a target plant to yield transgenic plants, regarded as one of the most valuable systems to produce human therapeutic antibodies of various ideal traits. For successful production of antibodies, careful consideration to select plant species is a must because every plant species possesses its own physiological and physiochemical characteristics that affect the expression and glycosylation of recombinant glycoproteins. Plants have been chosen as a promising biofactory system to produce antibodies in a large scale because of their high production capacity with affordable cost, higher scalability, reduced screening costs for pathogens, low downstream processing requirements, and easy growth under containment conditions. Many plant-derived mAbs have been produced in large manufacturing scales under the cGMP regulations and have been shown to meet US Food and Drug Administration (FDA) quality standards in identity, purity, and potency. Many of these have shown the proper assembly, effective *in vitro* neutralization, and potent *in vivo* efficacy in animal models. Over the past decades, the FDA has approved more than a dozen mAbs for the treatment of various malignancies, and hundreds of companies have been motivated to be involved in the development of these molecules. Current advancements in genetic engineering, glycoengineering, and other posttranslational modifications have made new strides to provide additional advantages far beyond the traditional benefits of high scalability, economic feasibility, and increased safety, and it is hoped that an excellent platform for the future development of a safer monoclonal antibody is established.

## Figures and Tables

**Table 1:** Examples of plant production systems used for cancer immunotherapy [28].

Malignancy	Antigen/plant system used
Hepatitis B virus-induced hepatocellular carcinoma	HBsAg expressed in transgenic plants
Hepatitis C virus-induced hepatocellular carcinoma	E7 protein expressed in chloroplasts
Non-Hodgkin's lymphoma	Full IgG expressed in TMV-based expression system
Breast cancer	PVX nanoparticles expressing HER2 epitope
Solid tumors	PapMV nanoparticles
Lung melanoma	CPMV nanoparticles
Solid tumors	TMV nanoparticles displaying cRGD

CPMV: cowpea mosaic virus; HBsAg: hepatitis B virus surface antigen; PapMV: papaya mosaic potexvirus; PVX: potato virus X; TMV: tobacco mosaic virus.
